# Role of ER Stress in Ventricular Contractile Dysfunction in Type 2 Diabetes

**DOI:** 10.1371/journal.pone.0039893

**Published:** 2012-06-29

**Authors:** Akifumi Takada, Takayuki Miki, Atsushi Kuno, Hidemichi Kouzu, Daisuke Sunaga, Takahito Itoh, Masaya Tanno, Toshiyuki Yano, Tatsuya Sato, Satoko Ishikawa, Tetsuji Miura

**Affiliations:** 1 Second Department of Internal Medicine, Sapporo Medical University School of Medicine, Sapporo, Japan; 2 Department of Pharmacology, Sapporo Medical University School of Medicine, Sapporo, Japan; 3 Department of Cellular Physiology and Signal Transduction, Sapporo Medical University School of Medicine, Sapporo, Japan; UAE University, Faculty of Medicine & Health Sciences, United Arab Emirates

## Abstract

**Background:**

Diabetes mellitus (DM) is associated with an increased risk of ischemic heart disease and of adverse outcomes following myocardial infarction (MI). Here we assessed the role of endoplasmic reticulum (ER) stress in ventricular dysfunction and outcomes after MI in type 2 DM (T2DM).

**Methodology and Principal Findings:**

In hearts of OLETF, a rat model of T2DM, at 25∼30 weeks of age, GRP78 and GRP94, markers of ER stress, were increased and sarcoplasmic reticulum calcium ATPase (SERCA)2a protein was reduced by 35% compared with those in LETO, a non-diabetic control. SERCA2a mRNA levels were similar, but SERCA2a protein was more ubiquitinated in OLETF than in LETO. Left ventricular (LV) end-diastolic elastance (Eed) was higher in OLETF than in LETO (53.9±5.2 vs. 20.2±5.6 mmHg/µl), whereas LV end-systolic elastance and positive inotropic responses to β-adrenergic stimulation were similar in OLETF and LETO. 4-Phenylbutyric acid (4-PBA), an ER stress modulator, suppressed both GRP up-regulation and SERCA2a ubiquitination and normalized SERCA2a protein level and Eed in OLETF. Sodium tauroursodeoxycholic acid, a structurally different ER stress modulator, also restored SERCA2a protein level in OLETF. Though LV dysfunction was modest, mortality within 48 h after coronary occlusion was markedly higher in OLETF than in LETO (61.3% vs. 7.7%). Telemetric recording showed that rapid progression of heart failure was responsible for the high mortality rate in OLETF. ER stress modulators failed to reduce the mortality rate after MI in OLETF.

**Conclusions:**

ER stress reduces SERCA2a protein via its augmented ubiquitination and degradation, leading to LV diastolic dysfunction in T2DM. Even at a stage without systolic LV dysfunction, susceptibility to lethal heart failure after infarction is markedly increased, which cannot be explained by ER stress or change in myocardial response to sympathetic nerve activation.

## Introduction

Diabetes mellitus (DM) is associated with an increased risk of coronary artery disease and of adverse outcomes following myocardial infarction (MI), including death and recurrent myocardial infarction (MI) [1]. The risk of non-ischemic heart failure is also increased by DM [1], [2]. Multiple factors, including impaired Ca^2+^ homeostasis, increased oxidative stress and mitochondrial dysfunction, have been suggested to contribute to DM-induced myocardial dysfunction, so-called “DM cardiomyopathy” [2]. However, severity and characteristics of ventricular dysfunction in diabetic hearts are different depending on the type and duration of DM and animal species [2]–[7]. In fact, effects of DM on mortality rate after MI were discrepant in earlier laboratory investigations, and the mechanisms of reduced post-MI survival in DM are still uncertain [5], [6], [8], [9].

In the present study, we aimed to determine the role of endoplasmic reticulum (ER) stress in ventricular dysfunction at an early stage of DM cardiomyopathy and outcomes after MI in type 2 DM (T2DM). We focused on ER stress in the myocardium for three reasons. First, accumulating evidence indicates significant roles of ER stress in the pathophysiology of DM [10], [11]. Second, it has been shown that sarcoplasmic reticulum (SR) plays a pivotal role in Ca^2+^ handling and that SR dysfunction leads to contractile dysfunction of the heart [12]. Third, recent studies showed that increased ER stress contributes to apoptosis of cardiomyocytes in streptozotocin-induced DM [13] and to impairment of cardioprotective signaling in rat model of T2DM [14]. DM has been shown to reduce SR calcium ATPase (SERCA)2a expression in the heart, which was causally related with contractile dysfunction in earlier studies [15]–[20]. We used Otsuka Long-Evans-Tokushima Fatty rats (OLETF), an established model of spontaneously developing T2DM, at ages of 25∼30 weeks in this study. Our previous studies [14], [21] have shown that OLETF at these ages have the metabolic phenotype of hyperinsulinemic T2DM and increased levels of GRP78 and GRP94, ER stress markers, in the myocardium. Acute responses of heart rate and blood pressure to coronary artery occlusion in OLETF were not significantly different from those in their non-diabetic controls, Long-Evans-Tokushima Fatty rats (LETO) [14], [21], indicating absence of severe loss of ventricular contractile reserve in OLETF at ages of 25∼30 weeks old. In contrast, significant reduction in ventricular contractility has been reported for OLETF at 70∼80 weeks of age [18]. Results of the present study suggest that ER stress-mediated enhancement of SERCA2a ubiquitination is responsible for down-regulation of SERCA2a protein expression and diastolic dysfunction of the heart. Although ventricular contractility at rest and upon β-adrenergic stimulation was preserved in 25∼30-week-old OLETF, mortality rate after MI was eight-fold higher than that in the non-diabetic controls due to an ER stress-independent mechanism.

## Results

### Hemodynamics and Blood Glucose Level in a Conscious State

As in our previous studies [14], [21], OLETF had larger body weight and higher blood glucose level than those of LETO, indicating a phenotype of T2DM (Table 1). Mean blood pressure, but not heart rate, in a conscious state was higher by approximately 20 mmHg in OLETF than in LETO. Pretreatment with 4-phenylbutyric acid (4-PBA), an ER stress modulator [22], did not modify body weight or hemodynamics but reduced blood glucose level by 36% in OLETF, whereas none of these parameters were changed by pretreatment with cyclosporine A, an inhibitor of calcineurin.

**Table 1:** Body weight, hemodynamics and blood glucose level.

	LETO	OLETF	OLETF	OLETF
			+4-PBA	+cyclosporine A
	(n = 14)	(n = 17)	(n = 15)	(n = 13)
Body weight (g)	542±7	641±13*	632±10*	657±9*
Blood glucose (mg/dl)	137±6	274±14*	175±11†	258±21*
Heart rate (beats/min)	345±9	339±6	327±6	330±5
Mean blood pressure (mmHg)	92±3	112±2*	117±4*	113±2*

Values are means±SEM.

* P<0.05 vs. LETO.

† P<0.05 vs. OLETF.

4-PBA  = 4-phenylbutyric acid.

### LV Functions and Response to β-adrenoceptor Stimulation

Under pentobarbital anesthesia, heart rate was lower and left ventricular (LV) end-systolic pressure was higher in OLETF than in LETO (Table 2). Since the difference in heart rate was undetectable under conscious state (Table 1), the anesthesia might have unmasked alterations in heart rate regulation by the autonomic nervous system in OLETF. LV wall thickness determined by echocardiography was significantly larger in OLETF than in LETO (Table 3). However, there were no differences in LV luminal dimension and fractional shortening measured by echocardiography between OLETF and LETO groups.

**Table 2:** Summary data from pressure-volume relationships.

	LETO	OLETF	OLETF+4-PBA
	(n = 5)	(n = 6)	(n = 6)
Body weight (g)	533±17	605±26*	624±14*
Heart weight (g)	1.53±0.05	1.83±0.11*	1.74±0.02
Heart weight/Body weight (%)	0.29±0.01	0.30±0.01	0.28±0.01
HR (beats/min)	391±8	341±16*	327±9*
LVESP (mmHg)	100.5±1.6	133.2±3.2*	136.2±9.1*
LVEDP (mmHg)	10.4±3.0	5.5±1.8	4.6±2.3
LVEDV (ml)	0.14±0.012	0.17±0.013	0.18±0.019
LVESV (ml)	0.06±0.005	0.09±0.012	0.07±0.013
SV (ml)	0.08±0.009	0.09±0.017	0.11±0.008*
LVEF (%)	56.3±3.4	50.8±4.2	62.4±3.8
CO (ml/min)	30.6±3.1	29.0±2.4	37.3±3.3
LV dP/dtmax (mmHg/s)	6665±805	7469±356	8660±555
LV dP/dtmin (mmHg/s)	−6824±545	−8633±561*	−9428±276*
tau (ms)	10.2±1.1	9.7±1.7	10.3±1.2
Ees (mmHg/ml)	3392±567	3397±418	2748±356
Eed (mmHg/ml)	20.2±5.6	53.9±5.2*	24.2±6.0
Vp (ml)	0.19±0.02	0.20±0.01	0.20±0.01

Values are means±SEM. *P<0.05 vs. LETO. HR =  heart rate; LVESP =  left ventricular end-systolic pressure; LVEDP =  left ventricular end-diastolic pressure; LVEDV =  left ventricular end-diastolic volume; LVESV =  left ventricular end-systolic volume; LVEF =  left ventricular ejection fraction; CO =  cardiac output; LV dP/dtmax and dP/dtmin =  left ventricular maximal slope of the systolic pressure increment and the diastolic pressure decrement, respectively; tau =  time constant of left ventricular pressure decay; Ees =  end-systolic elastance of left ventricle; Eed =  end-diastolic elastance of left ventricle; Vp =  parallel conductance volume.

**Table 3:** Echocardiographic data at baseline.

	LETO	OLETF	OLETF	OLETF
			+4-PBA	+cyclosporine A
	(n = 13)	(n = 25)	(n = 13)	(n = 14)
LVEF (%)	80.9±0.7	79.9±0.7	80.6±0.6	80.5±0.6
%FS	44.7±0.7	43.9±0.7	44.5±0.6	44.4±0.6
IVST (mm)	1.56±0.07	1.86±0.05*	1.80±0.06*	1.80±0.06*
PWT (mm)	2.05±0.08	2.22±0.05	2.12±0.04	2.32±0.06*
LVEDD (mm)	7.73±0.25	8.01±0.12	8.11±0.10	8.13±0.15
LVESD (mm)	4.31±0.17	4.48±0.08	4.49±0.07	4.46±0.13
LVEDV (ml)	1.05±0.08	1.14±0.04	1.17±0.04	1.12±0.07
LVESV (ml)	0.21±0.02	0.23±0.01	0.23±0.01	0.23±0.19

Values are means±SEM. *P<0.05 vs. LETO. 4-PBA = 4-phenylbutyric acid; LVEF =  left ventricular ejection fraction; %FS =  fractional shortening; IVST =  interventricular septal thickness; PWT =  posterior wall thickness; LVEDD =  left ventricular end-diastolic dimension; LVESD =  left ventricular end-systolic dimension; LVEDV =  left ventricular end-diastolic volume; LVESV =  left ventricular end-systolic volume.

**Figure 1:**
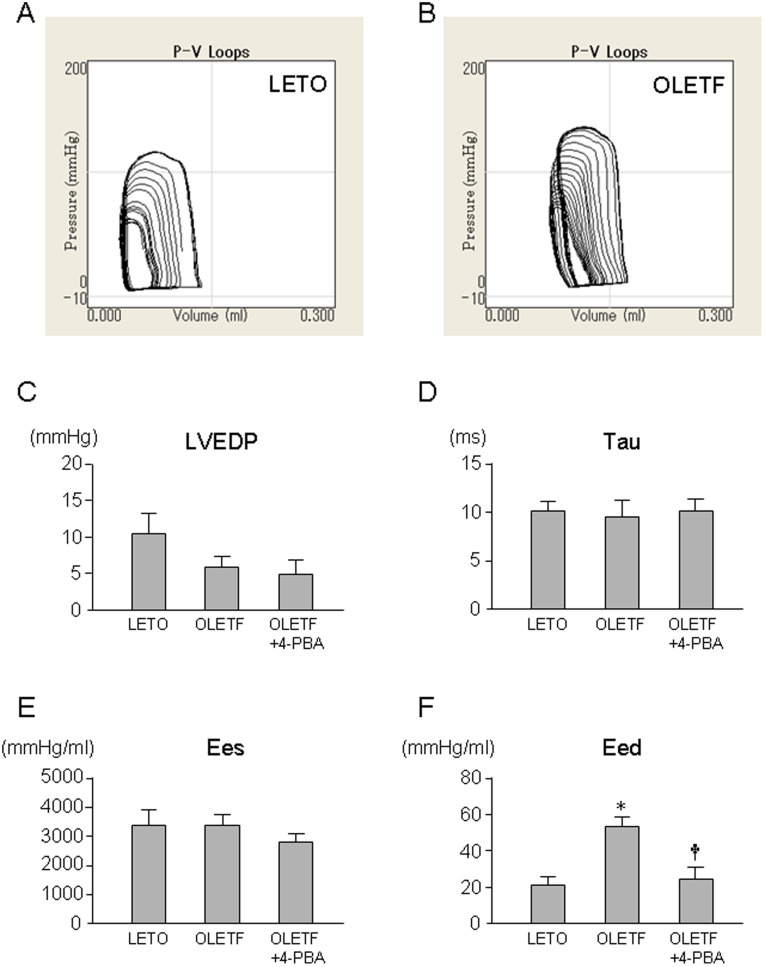
LV functions determined by pressure-volume relationships. Representative examples of pressure-volume relationships in LETO (A) and OLETF (B) and summary data of LVEDP (C), tau (D), LV Ees (E), and LV Eed (F) are shown. *p<0.05 vs. LETO, †p<0.05 vs. OLETF. n = 5∼6. LVEDP =  left ventricular end-diastolic pressure; tau =  time constant of left ventricular pressure decay; Ees =  end-systolic elastance; Eed =  end-diastolic elastance; 4-PBA = 4-phenylbutyric acid.

In contrast to the reported reduction in LV end-systolic elastance (Ees) in OLETF at ages of 70∼80 weeks old [18], Ees and other indices of systolic functions determined by pressure-volume (P-V) loop analysis in 25∼30 week-old-OLETF were not significantly reduced compared with those in LETO. Treatment with 4-PBA or cyclosporine A and did not modify these parameters in OLETF (Tables 2 and 3). Other loading condition-dependent and independent parameters of systolic function were also similar among LETO, OLETF and OLETF treated with 4-PBA (Figure 1 and Table 2). Although values of time constant of LV pressure decay (tau) were similar in OLETF and LETO, end-diastolic elastance (Eed), slope of end-diastolic pressure-volume relationship, was significantly larger in OLETF than in LETO (53.9±5.2 vs. 20.2±5.6 mmHg/µl). Treatment with 4-PBA normalized Eed in OLETF (Eed = 24.2±6.0 mmHg/µl). LV dP/dtmin was paradoxically larger in OLTEF than in LETO, but the difference is attributable to different LV loading conditions indicated by differences in heart rate and LV end-systolic pressure between the two groups (Table 2).

**Figure 2:**
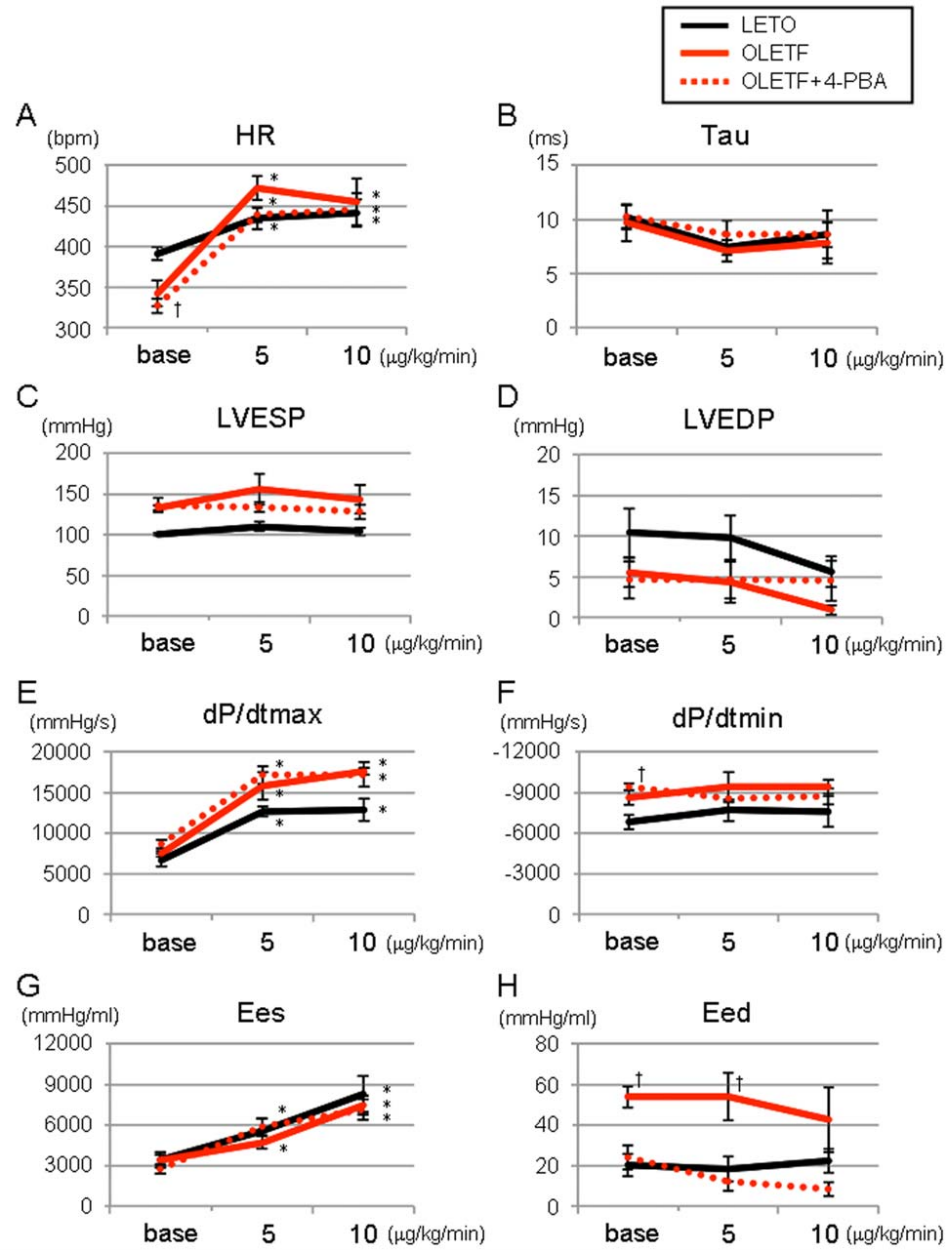
Responses of LV functions to β-adrenoceptor stimulation. Responses of heart rate (A), tau (B), LVESP (C), LVEDP (D), LVdP/dtmax (E), LVdP/dtmin (F), Ees (G) and Eed (H) to intravenous infusion of dobutamine are shown. *p<0.05 vs. Baseline (Base), †p<0.05 vs. LETO. n = 5∼6. tau =  time constant of left ventricular pressure decay; LVESP =  left ventricular end-systolic pressure; LVEDP =  left ventricular end-diastolic pressure; LVdP/dtmax =  left ventricular maximal slope of the systolic pressure increment; LVdP/dtmin =  left ventricular maximal slope of the diastolic pressure decrement; Ees =  end-systolic elastance; Eed =  end-diastolic elastance; 4-PBA = 4-phenylbutyric acid.

Infusion of dobutamine (5∼10 µg/kg/min) increased heart rate, LVdP/dtmax and Ees in LETO. These positive inotropic responses to dobutamine were similarly induced in OLETF with or without 4-PBA pretreatment (Figure 2). Responses of tau and Eed to dobutamine were also similar in the three study groups.

### Expression of ER Stress Markers and Sarcoplasmic Proteins: Immunoblot and qRT-PCR Data

**Figure 3:**
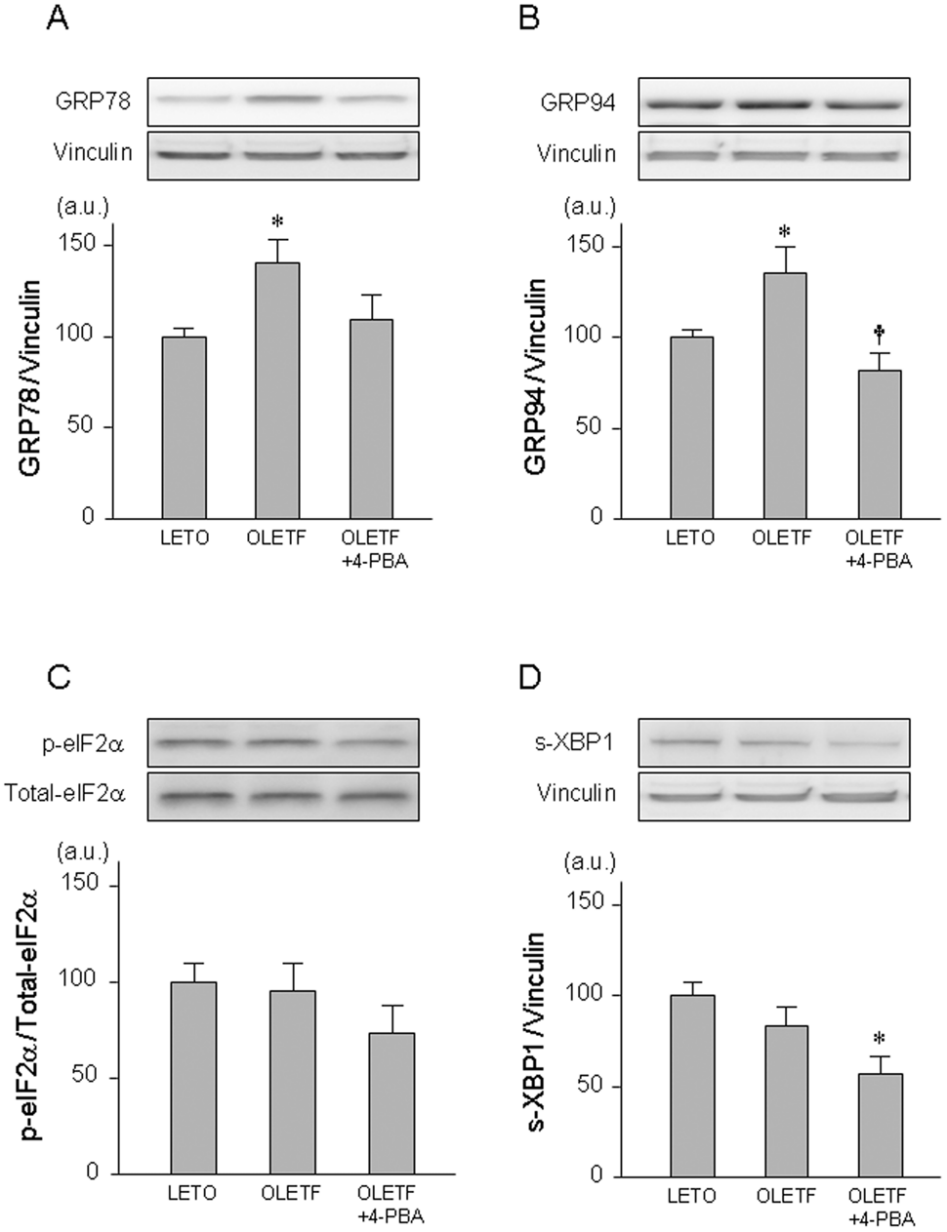
ER stress-related proteins in OLETF and LETO. Representative blots (upper panel) and summary of data (lower panel) from immnoblotting of GRP78 (A), GRP94 (B), phospho-eIF2α (C), and spliced XBP1 (D) are shown. *p<0.05 vs. LETO. †p<0.05 vs. OLETF. n = 6∼8. GRP78 =  glucose-regulated protein 78; GRP94 =  glucose-regulated protein 94; eIF2α =  eukaryotic initiation factor 2α; XBP-1 =  X-box binding protein-1; 4-PBA = 4-phenylbutyric acid.

Consistent with our previous reports, myocardial levels of GRP78 and GRP94, markers of ER stress, were significantly higher in OLETF than in LETO (Figure 3). Like sodium tauroursodeoxycholic acid (TUDCA), a chemical chaperone, in our previous study [14], 4-PBA normalized levels of glucose-regulated protein (GRP)78 and GRP94 in OLETF in the present study. Levels of phospho-eIF2α and spliced X-box binding protein (XBP)-1 were similar in OLETF and LETO. Other ER stress-related proteins, including IRE1α, PERK and ATF6, could not be detected in the rat myocardium by immunoblotting in the present experiments (data not shown), though their mRNAs levels detected by DNA microarray analysis were similar in LETO and OLETF in our preliminary experiments.

**Figure 4:**
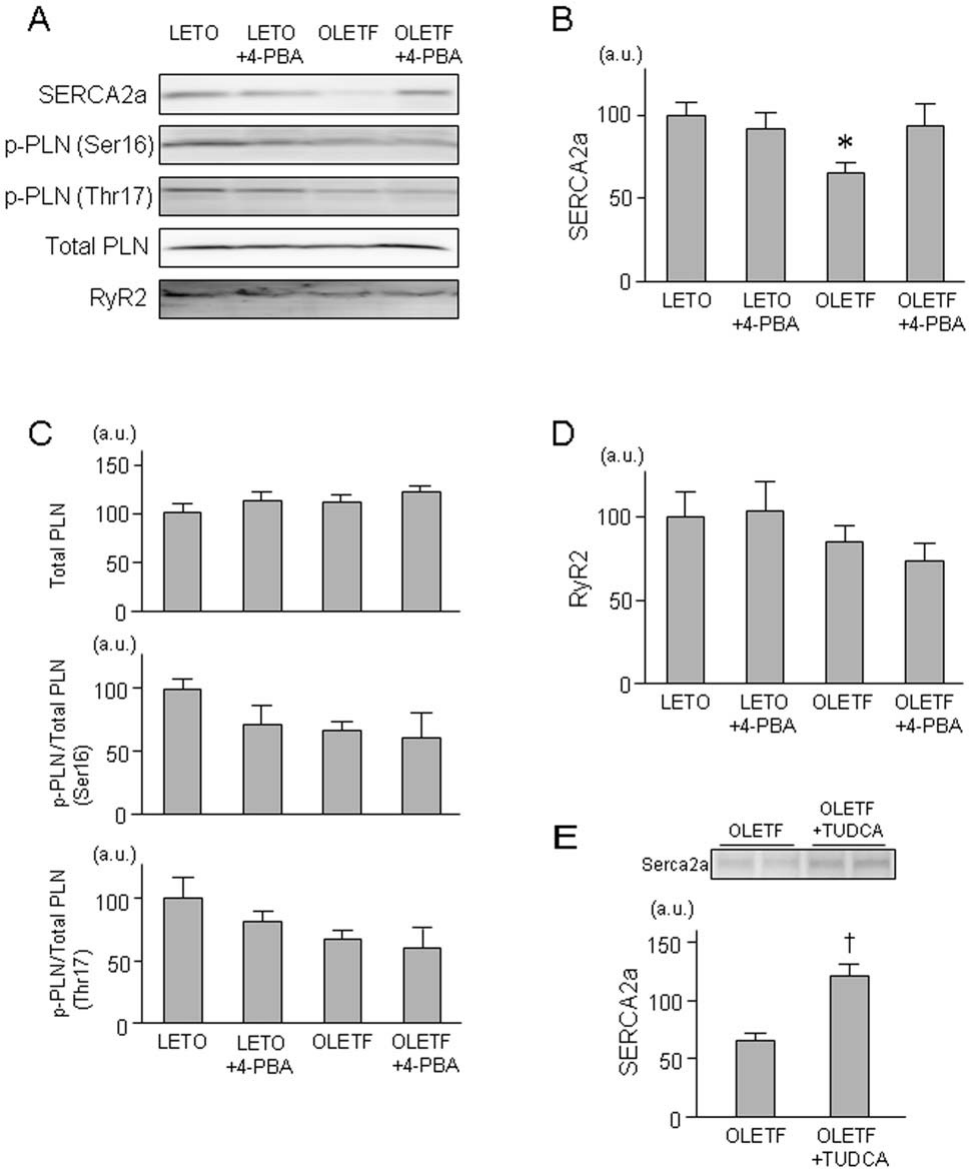
Sarcoplasmic proteins in OLETF and LETO. Representative blots (A) and summary of data from immnoblotting of SERCA2a (B), total PLN, and phospho-PLN at Ser16 and Thr17 (C), and RyR2 (D) are shown. n = 8∼15. Representative blots (upper panel) and summary of data (lower panel) from immnoblotting of SERCA2a (E) in a *post-hoc* experiment (n = 5) is also shown. *p<0.05 vs. LETO. † p<0.05 vs. OLETF. SERCA2a =  sarcoplasmic reticulum calcium ATPase 2a; PLN =  phospholamban; RyR =  ryanodine receptor; 4-PBA = 4-phenylbutyric acid; TUDCA =  sodium tauroursodeoxycholic acid.

Protein level of SERCA2a was significantly reduced by 35% in OLETF, and this reduction was restored by treatment with 4-PBA (Figure 4AB). Phospholamban level and its phosphorylation were not statistically significant (Figure 4AC). Protein levels of ryanodine receptor 2 were also similar in the study groups (Figure 4D). A *post-hoc* experiment using TUDCA confirmed that the reduction of SERCA2a protein in OLETF was restored by suppression of ER stress (Figure 4E).

**Figure 5:**
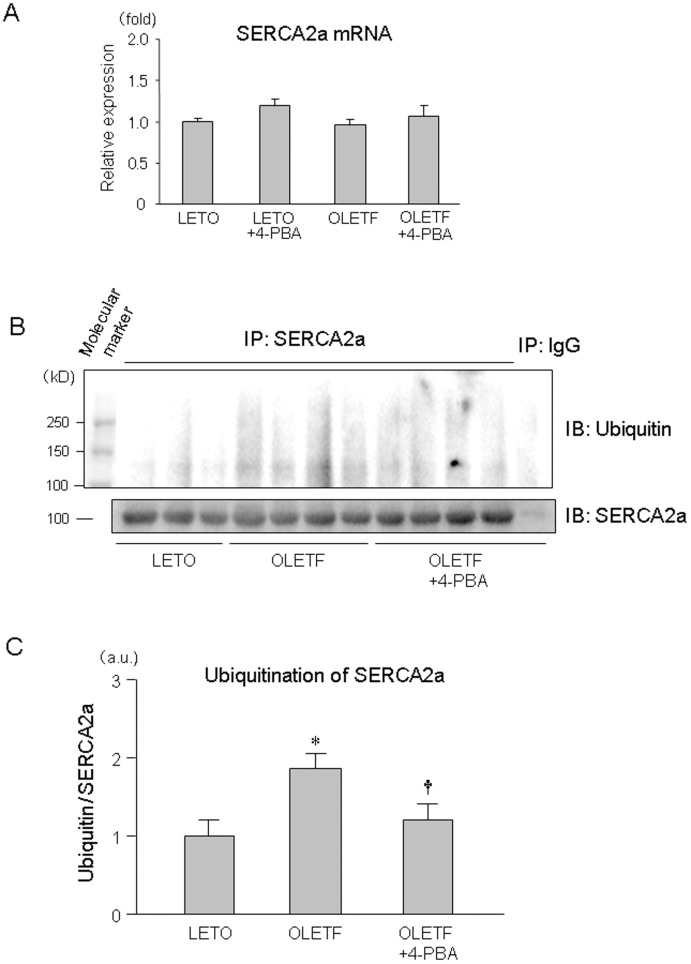
Alterations in mRNA expression and ubiquitination of SERCA2a in OLETF. Summary data of SERCA2a mRNA (A), representative blot (B) and summary data (C) of ubiquitination of SERCA2a protein are shown. *p<0.05 vs. LETO, †p<0.05 vs. OLETF. n = 5. SERCA2a =  sarcoplasmic reticulum calcium ATPase 2a; 4-PBA = 4-phenylbutyric acid.

As for the mechanism by which SERCA2a protein level is reduced in OLETF, we first determined SERCA2a mRNA levels in OLETF and LETO. However, there was no significant difference between SERCA2a mRNA levels in LETO and OLETF with and without 4-PBA (Figure 5A). Second, we examined whether activity of the ubiquitination-proteasome pathway is enhanced in OLETF. As shown in Figure 5B and 5C, ubiquitination of SERCA2a protein was significantly increased by 85% in OLETF compared with that in LETO. Interestingly, this enhanced ubiquitination of SERCA2a protein in OLETF was attenuated by 4-PBA.

To examine the possibility that O-glycosamine-N-acetylation (O-GlcNAcylation) of SERCA2a is a mechanism of its hyperubiquitination in OLETF, we determined level of N-acetylglucosamine (GlcNAc) in immunoprecipitated SERCA2a by using anti-GlcNAc antibody (RL-2). However, there was no significant difference between GlcNAc levels in LETO, OLETF and 4-PBA-treated OLETF (data not shown).

### Ventricular Function and Mortality After MI

**Figure 6:**
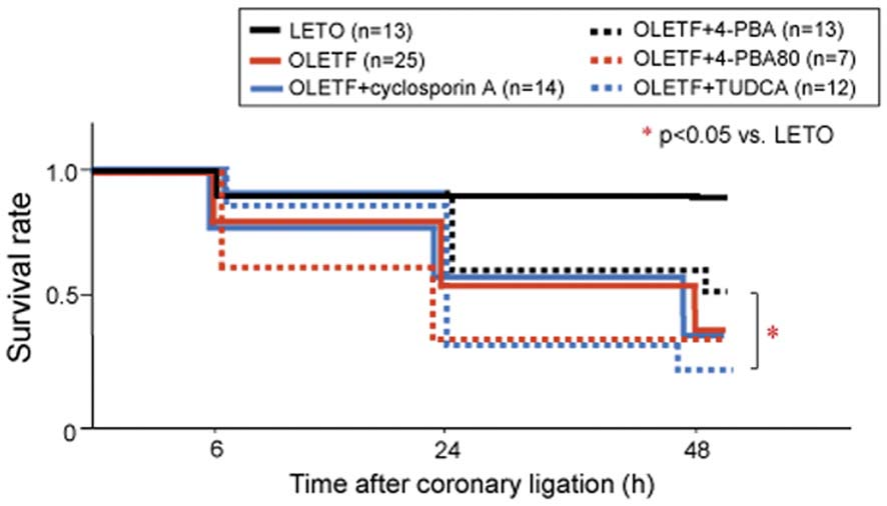
Survival after myocardial infarction. Kaplan-Meier survival analysis of rats with acute myocardial infarction. *p<0.05 vs. LETO. 4-PBA  =  pretreatment with 4-PBA at dose of 40 mg/kg/day; 4-PBA80 =  pretreatment with 4-PBA at a dose of 80 mg/kg/day; Cyclosporine A  =  pretreatment with cyclosporine A at dose of 20 mg/kg/day; TUDCA =  pretreatment with sodium tauroursodeoxycholic acid (100 mg/kg/day). All agents were administered intraperitoneally for 7 days before infarct experiments. Experiments using TUDCA and a higher dose of 4-PBA (i.e., 4-PBA80 group) were additionally performed as *post-hoc* experiments.

Echocardiography showed that thickening of the LV anterior wall was severely impaired 1 h after coronary ligation in all animals. There were no significant differences in LV dimension, fractional shortening and size of akinetic areas between the treatment groups, indicating that there was no inter-group difference in infarct sizes (Table 4). The survival rate within 48 h after MI was significantly lower in control OLETF than in LETO (Figure 6). Telemetric monitoring of ECG and blood pressure after MI in a group of OLETF (n = 5) showed that progressive hypotension preceded bradycardia and cardiac arrest, and ventricular tachycardia or fibrillation preceding hypotension was not observed in any cases. The findings indicate that progression of heart failure, but not ventricular arrhythmias, was responsible for the increased post-MI mortality. The survival rate in OLETF was not significantly improved by either 4-PBA or cyclosporine A. To confirm the lack of a significant effect of ER stress suppression, we additionally examined the effect of TUDCA in a *post-hoc* series of experiments. TUDCA also failed to improve survival rates after MI (Figure 6). In additional series of *post-hoc* series of MI experiments, we doubled the dose of 4-PBA in OLETF (n = 7), but the mortality rate within 48 h after MI was not reduced (Figure 6). On the other hand, only one of five LETO pretreated with 4-PBA died within 48 h after MI, indicating that 4-PBA alone did not change the post-MI mortality rate.

**Table 4:** Echocardiographic data at 1 hr after coronary ligation.

	LETO	OLETF	OLETF	OLETF
			+4-PBA	+cyclosporine A
	(n = 12)	(n = 22)	(n = 13)	(n = 13)
LVEF (%)	36.3±2.2	39.3±1.2	41.3±1.6	40.9±0.9
%FS	15.2±1.1	16.6±0.6	17.6±0.8	17.4±0.5
IVST (mm)	1.52±0.09	1.74±0.05	1.82±0.06*	1.71±0.08
PWT (mm)	1.73±0.09	1.96±0.04	2.06±0.05*	1.97±0.11
LVEDD (mm)	7.78±0.41	8.12±0.16	7.48±0.29	7.81±0.25
LVESD (mm)	6.61±0.34	6.78±0.16	6.27±0.27	6.45±0.21
LVEDV (ml)	1.11±0.17	1.19±0.06	1.01±0.09	1.07±0.09
LVESV (ml)	0.70±0.09	0.72±0.05	0.61±0.07	0.63±0.06
akinetic area (%)	37±3	38±2	40±3	39±1

Values are means±SEM. *P<0.05 vs. LETO. Abbreviations: See Table 2. Echocardiographic data was not acquired from 1 rat in LETO, 3 in OLETF and 1 in OLETF+cyclosporine A because of the poor quality of echocardiographic images.

In agreement with echocardiographic data, histological analysis confirmed that there was no significant difference between the five study groups in mean sizes of infarct, ranging from 38.2 to 41.4% of the LV. The numbers of Factor VIII-positive vessels in the infarct areas and border-zone areas were similar in the groups (data not shown).

## Discussion

Reduction of SERCA2a protein by diabetes has been known for some time, but its mechanism remains unclear. The mRNA level of SERCA2a has been reported to be reduced in some, but not all, models of diabetes [15]–[20]. A plausible explanation for the different findings in previous studies is the possibility that impaired transcription of the SECRA2a gene is a late event in the natural history of DM and is preceded by reduction in SERCA2a protein by non-transcriptional mechanisms. As for the mechanism of DM-induced suppression of SERCA2a transcription, O-GlcNAcylation of nuclear protein has been suggested by the results of a study by Clarke et al. [23]. They showed that hyperglycemia reduced both mRNA level and promoter activity of SERCA2a in isolated cardiomyocytes and that the effect of hyperglycemia on SERCA2a was attenuated by overexpression of O-GlcNAcase and mimicked by overexpression of O-GlcNAc-transferase. On the other hand, how SERCA2a protein level in diabetic hearts is reduced in the absence of change in SERCA2a mRNA level [17] was unclear. In the present study, treatment with both 4-PBA and TUDCA restored SERCA2a protein level in OLETF, and comparable results were recently reported for obese ob/ob mice by Ceylan-lsik et al. [24] using TUDCA. Furthermore, the present study showed for the first time that SERCA2a in OLETF is hyperubiquitinated and that the hyperubiquitination was significantly attenuated by 4-PBA (Figure 5). Taken together, the findings suggest that ER stress-mediated hyperubiquitination and subsequent degradation of SERCA2a is a mechanism of DM-induced down-regulation of SERCA2a in an early phase of DM (i.e., a phase preceding the down-regulation of SERCA2a mRNA level).

Mechanism by which ER stress induces hyperubiquitination of SERCA2a in OLETF remains unclear. We hypothesized that O-GlcNAcylation of SERCA2a primes hyperubiquitination of this protein in diabetes based on a recent report that hyperubiquitination of atheroprotective protein A20 in diabetic smooth muscle cells is mediated by O-GlcNAcylation [25]. However, this hypothesis was argued against by the results that levels of O-GlcNAcylation of SERCA2a were similar in LETO, OLETF and 4-PBA-treated OLETF. As another type of post-translational modification, formation of advanced glycation end-product (AGE) on SERCA has been reported in streptozotocin-induced diabetic rats [17]. However, relationship between AGE and ubiquitination/proteasome pathway is currently unknown and warrants further investigation.

As observed in patients with an early stage of DM cardiomyopathy [26], [27], OLETF at ages of 25∼30 weeks showed significant impairment of diastolic LV function without systolic dysfunction (Figure 1). Involvement of ER stress in the diastolic dysfunction was indicated by results showing that 4-PBA normalized Eed in OLETF. In ventricular dysfunction induced by diabetes, multiple mechanisms, including down-regulation of SERCA2a, reduced phosphorylation of phospholamban and leakage of Ca^2+^ from SR, and increased collagen deposition in the extracellular matrix [28]–[30], have been proposed. In the present study, 4-PBA restored SERCA2a protein level, while levels of phospholamban phosphorylation at Thr17 and Ser16 and ryanodine receptor protein were not modified by 4-PBA. There was no discernible difference in the extent of interstitial fibrosis between OLETF and LETO (data not shown). Thus, normalization of Eed by 4-PBA is likely to be due to normalized SERCA2a expression and Ca^2+^ uptake into the SR during diastole, though the possibility of involvement of other 4-PBA targets cannot be excluded.

Down-regulation of SERCA2a occurs in not only diabetic hearts but also other types of heart failure [12]. Gene therapy using an adenovirus or adenovirus-associated virus has been designed to restore SERCA2a protein level in failing hearts for improving ventricular function and/or ventricular arrhythmias. In fact, several groups have reported that such SERCA2a gene therapy significantly attenuated systolic dysfunction in diabetic hearts [18], [31]–[33]. Although the ventricular dysfunction was very modest in the present model of T2DM, a chemical chaperone successfully restored SERCA2a level and diastolic function. Recently, systolic ventricular dysfunction in obese diabetic mice (obese ob/ob mice) was shown to be improved by TUDCA, indicating that the role of ER stress is not limited to diastolic dysfunction [24]. Furthermore, increase in ER stress has been demonstrated in heart failure induced by pressure overload and by coronary embolization [34]–[36], indicating a possible role of ER stress in the pathogenesis of heart failure in general. Nevertheless, chemical chaperones are clinically more feasible than viral vectors [37], and their effects on failing hearts warrant future investigations.

To our surprise, the mortality rate after MI was markedly increased in OLETF compared with that in LETO (Figure 6A), though there was only a modest difference in Eed and no difference in Ees or LV ejection fractions between OLETF and LETO. The increased mortality rate in OLETF is consistent with increased mortality rates in previous studies in which infarct size was larger than 20% of the left ventricle in different animal models of diabetes (i.e., Goto-Kakizaki rats, high fat diet-induced DM, streptozotocin-induced DM) [5], [6], [9]. However, the mechanism by which DM increases mortality during the acute phase of MI has not been explored. Telemetric analysis in the present study showed that progression of heart failure, not ventricular arrhythmia, was responsible for the higher mortality. As a possible reason for the high post-MI mortality in OLETF, we first postulated the possibility that compensatory increase in contraction of the non-ischemic region after MI is attenuated due to reduced Ca^2+^ store in the SR and/or autonomic dysfunction associated with DM. However, this possibility was not supported by results showing that responses of Ees, Eed and dP/dts to dobutamine infusion were similar in OLETF and LETO (Figure 1).

We then postulated that defective LV diastolic function or loss of cytoprotective signaling underlies the high post-MI mortality in OLETF. However, this hypothesis was argued against by results showing that 4-PBA, TUDCA or cyclosporine A did not improve survival of OLETF after MI, though doses of the chemical chaperones and cyclosporine A were sufficient for normalizing LV Eed in this study (Figure 1) and for repairing defective cardioprotective signaling, respectively, in OLETF [21]. One of remaining possible explanations for the high mortality in OLETF is increased volume retention by augmented activation of the renin-angiotensin system (RAS) after MI in OLETF. To critically test the hypothesis, a future study will be necessary for examining differences in time courses of the RAS activation, fluid balance and hemodynamic parameters after MI between OLETF and LETO.

In conclusion, ER stress reduces SERCA2a protein via its augmented ubiquitination and degradation, leading to LV diastolic dysfunction in T2DM. Even at a stage of modest diastolic LV dysfunction, susceptibility of diabetic hearts to lethal heart failure after MI is substantially increased, and the susceptibility cannot be explained by reduced SERCA2a expression, up-regulated calcineurin activity or impaired myocardial response to sympathetic nerve activation.

## Materials and Methods

The present study was conducted in strict accordance with The Guide for the Care and Use of Laboratory Animals published by the US National Institutes of Health (NIH publication No. 85–23, revised 1996) and was approved by the Animal Use Committee of Sapporo Medical University (#08-148_10-083).

### Animals

LETO and OLETF at ages of 25∼30 weeks were kindly provided by the Tokushima Research Institute of Otsuka Pharmaceutical, Tokushima, Japan. LETO and OLETF were pretreated with saline or 4-phenylbutyric acid (4-PBA, 40 mg/kg/day, i.p.), an ER stress modulator, for 1 week before the experiments. 4-PBA acts as a chemical chaperone, assisting with protein folding and thus reducing ER stress [22]. In MI experiments, LETO received saline and OLETF received saline, 4-PBA or cyclosporine A (20 mg/kg/day, i.p.) for 1 week before induction of MI. The same dose of cyclosporine A has been shown by our previous study to repair defects in cytoprotective signaling by up-regulated calcineurin in OLETF [21]. To confirm the results in 4-PBA-treated OLETF, a study group was added *post-hoc* by use of a structurally different chemical chaperone, sodium tauroursodeoxycholic acid (TUDCA) [14], [38]. TUDCA at a dose of 100 mg/kg/day (i.p.) was administered to an additional group of OLETF for 1 week before the experiment. As a *post-hoc* series of MI experiments, we examined the effect of 4-PBA (40 mg/kg/day, i.p. for 7 days) in LETO and a higher dose of 4-PBA (80 mg/kg/day, i.p. for 7 days) in OLETF.

### Measurements of Baseline Heart Rate and Blood Pressure

Systemic blood pressure and pulse rate were measured in a conscious state using a tail-cuff system (BP-98A, Softran, Tokyo, Japan). Rats were placed in a restrainer for 15 min, and then a cuff was attached to their tail and blood pressure and heart rate were measured.

### Echocardiography

Each rat was lightly sedated with 3–4% isoflurane, and then the chest was shaved and the rat was placed in the supine position. Echocardiography was performed at baseline and 1 h after induction of MI using a 11.5 MHz transducer connected to a Vivid-i Cardiovascular Ultrasound System (GE Medical, Milwaukee, USA). M-mode and 2-dimensional images at the papillary muscle level were acquired with a frame rate of 88.9/sec. Interventricular septal thickness (IVST), posterior wall thickness (PWT), LV end-diastolic dimension (LVEDD) and LV end-systolic dimension (LVESD) were measured. LV fractional shortening (FS) was calculated by the following formula: FS (%)  =  (LVEDD-LVESD)/LVEDD.

### P-V Loop Analyses

Rats were anesthetized with pentobarbital sodium (40 mg/kg, i.p.) and the adequacy of anesthesia was confirmed by disappearance of eyelid reflex, corneal reflex, loss of muscular tone, and no response to surgical manipulation. The level of anesthesia was continuously monitored during the experiment and an additional dose of pentobarbital (10 mg/kg, i.v.) was administered when necessary. After confirmation of the adequacy of anesthesia, rats were intubated and ventilated with a rodent respirator (model 683, Harvard Apparatus, South Natick, MA). The LV P-V relationship was determined by the method previously reported [39], [40]. A 3-F catheter-tip micromanometer (Miller Instruments, Houston, TX) was inserted into the LV cavity through the right carotid artery. An incision over the xyphoid was made and the diaphragm was cut to expose the apex of the heart, and then a conductance catheter with a 2-mm inter-electrode distance (Unique Medical, Tokyo, Japan) was inserted into the LV cavity through the apex. The distal driving electrode and proximal electrode were positioned at the level of the aortic valve and near the apex, respectively. To obtain LV P-V loops, we used a string-occluder to gradually occlude the inferior vena cava until LV end-diastolic volume (LVEDV) slightly decreased. The respirator was turned off during data acquisition to avoid respiratory-mediated hemodynamic fluctuation. For measurement of a parallel conductance volume, 0.02 mL of 5% NaCl solution was injected into the pulmonary artery, which transiently changed LV blood conductivity. The LV conductance volume was calculated by subtraction of the parallel conductance volume from the actual measured volume. Finally, blood sample conductivity was measured using a small cuvette. Integral 3 (Unique Medical) was used to store data from the conductance catheter and tip-manometer and to calculate the systolic and diastolic functional parameters, including Ees. To assess the possibility that myocardial response to β-adrenoceptor activation is modified in OLETF, we determined hemodynamic responses to dobutamine. Rats received infusion of dobutamine (5 and 10 µg/kg/min) for 4 min, during which time heart rate, arterial blood pressures, LV pressures, and LVdP/dt were continuously recorded as described above.

### Immunoblotting Study

Rats were anesthetized and ventilated as in LV P-V loop analysis experiments. Under anesthesia, hearts of the rats were excised and perfused with non-circulating Krebs-Henseleit buffer (NaCl 118.5, KCl 4.7, MgSO_4_ 1.2, KH_2_PO_4_ 1.2, NaHCO_3_ 24.8, CaCl_2_ 2.5 and glucose 10 mmol/L) at a pressure of 75 mmHg as previously reported [41]. The buffer was continuously gassed with 95% O_2_/5% CO_2_, and the temperature of the perfusate was maintained at 38°C. Myocardial tissues (0.8∼1.0 g) were sampled from the left ventricle and immediately frozen in liquid nitrogen and stored at −80°C until use for biochemical analysis. Total homogenates or sarcoplasmic reticulum fractions were prepared for immunoblotting. The sarcoplasmic reticulum preparation was isolated by the method previously described [42]. Tissue samples were homogenized in ice-cold buffer containing 10 mM NaHCO_3_, 5 mM NaN_3_, and 15 mM Tris-HCl (pH 6.8). The homogenate was centrifuged at 10,000 g for 20 min at 4°C to obtain the supernatant. The supernatant was further centrifuged at 40,000 g for 45 min at 4°C. The pellet was suspended in a buffer containing 0.6 M KCl and 20 mM Tris-HCl (pH 6.8) and then recentrifuged at 40,000 g for 45 min at 4°C. The final pellet was suspended in a mixture of 250 mM sucrose and 10 mM histidine (pH 7.0) and used for immunoblotting. All solutions contained 0.1% phenylmethylsulfonyl fluoride. Protein concentration was determined using a Bio-Rad Protein Assay Kit (Bio-Rad, Hercules, CA). Equal amounts of protein were analyzed by immunoblot assays using antibodies that recognize the following proteins: SERCA2a (Abcam, Cambridge, MA), Ser16-phosphorylated phospholamban (PLN) (Upstate, Temecula, CA), Thr17-phosphorylated PLN (Santa Cruz Biotechnology, Santa Cruz, CA), total PLN (Thermo Scientific, Rockford, IL), ryanodine receptor 2 (CHEMICON International, Temecula, CA), GRP78 (Cell Signaling Technology, Beverly, MA), GRP94 (Zymed, Carlsbad, CA), phosphor-eIF2α (Invitrogen, Camarillo, CA), total eIF2α (Cell Signaling Technology), spliced XBP-1 (Santa Cruz Biotechnology), phosphor-PERK (Cell Signaling Technology), total PERK (Rockland, Gilbertsville, PA), phosphor-IRE1a (Novus Biologicals, Littleton, CO), total IRE1a (Cell Signaling Technology), ATF6 (Imgenex, San Diego, CA) and vinculin (Sigma Aldrich, St. Louis, MO). PVDF membranes were first used for blotting of phosphorylated proteins. The blot was then stripped by a Re-Blot Western Recycling Kit (CHEMICON International, Temecula, CA) for re-blotting of total proteins and loading controls. Equal loading of protein onto each lane in the gel was confirmed later from comparable levels of vinculin protein and/or comparable densitometric levels of protein bands in the gels stained with Coomasie Brilliant Blue. Immunoblotted proteins were visualized by using an Immobilon Western detection kit (Millipore, Billerica, MA) and quantified by a lumino-image analyzer, LAS-2000mini (Fujifilm, Tokyo, Japan).

### Immunoprecipitation Study

An equal amount of protein (500 µg) from the sarcoplasmic reticulum fraction used in immunoblotting was solubilized in a CelLytic™ MT Mammalian Tissue Lysis/Extraction Reagent (Sigma Aldrich) including protease inhibitor cocktail (Nakalai Tesque, Kyoto, Japan) and a proteasome inhibitor, MG132, (10 µM) for 60 min at 4°C. The lysates were incubated for 30 min with protein A/G PLUS-agarose (Santa Cruz) to remove endogenous IgG. Then either 2 µl of the anti-SERCA2a antibody or normal mouse IgG was added to precleared lysates and incubated overnight at 4°C. Protein A/G PLUS-agarose slurry was added and rotary mixed for 60 min at 4°C. The slurry was centrifuged at 4°C for 5 min and washed 4 times by using PBS with protease inhibitor cocktail and MG132. Finally, the immunoprecipitated proteins were subjected to SDS-PAGE and transferred to PVDF membranes. Membranes were first used for blotting of the rabbit polyclonal ubiquitin antibody (1∶250, Santa Cruz, sc-9133) or anti-N-Acetylglucosamine (RL-2) (AbCam, Cambridge, MA) and then stripped and reblotted with the mouse monoclonal SERCA2a antibody (1∶2000).

### Quantitative Reverse Transcription Polymerase Chain Reaction (qRT-PCR)

Total RNA was isolated from rat LV tissues by using the RNeasy Fibrous Tissue Mini Kit (Qiagen, Valencia, CA) according to the manufacturer’s instructions. First-strand cDNA was synthesized using SuperScript III (Invitrogen, Carlsbad, CA). DNA amplification was performed in StepOneTM (Applied Biosystems, Foster City, CA) using the Power SYBR Green Master Mix (Applied Biosystems). The following primer pairs were used: 5′-GAAGCCATCAGCCAAGTCTC-3′ and 5′-CAGGGCCAATTAGAGAGCAG-3′ for Serca2a, 5′-TCACCACCATGGAGAAGGC-3′ and 5′-GCTAAGCAGTTGGTGGTGCA-3′ for GAPDH. All assays were performed in duplicate. Relative abundance of SERCA2a mRNA was examined by the standard curve method using serial cDNA dilution and was normalized by GAPDH as an internal control. GAPDH mRNA levels were similar in LETO, OLETF, 4PBA+LETO, and 4PBA+OLETF groups (data not shown).

### MI Study

OLETF and LETO were pretreated with saline, 4-PBA (40 mg/kg/day, i.p.), or cyclosporin A (20 mg/kg/day, i.p.) for 1 week before the experiments. Rats were anesthetized and ventilated as in LV P-V loop analysis experiments. After left thoracotomy, a coronary snare was placed around a marginal branch of the left coronary artery by using a 5–0 silk thread, and the coronary branch was then permanently ligated to induce myocardial infarction. The surgical wounds were repaired and the rats were returned to their cages for recovery. Ventricular dimensions and functions were assessed by echocardiography before and 1 h after coronary ligation. Survival of rats was checked at 6 h, 24 h and 48 h after myocardial infarction. To investigate the cause of death after infarction, a radio-telemetry device (Data Sciences International PhysioTel) was implanted in a group of OLETF (n = 5) as previously described [43]. In brief, the catheter part of a telemetry device, which has a pressure sensor at its tip, was inserted into the descending aorta for continuous non-tethered recording of pulsatile arterial blood pressure. Electrical leads from a telemetry device were placed in a modified lead II configuration by placing a negative electrode to the right of the manubrium and a positive electrode at the anterior axillary line along the 5^th^ intercostal space. Telemetry system implantation was performed one week prior to induction of myocardial infarction.

### Statistics

Data are expressed as means±SEM. The survival rates after MI were compared using Kaplan-Meier curves and log-rank statistics. Differences between treatment groups were tested by one-way ANOVA or two-way repeated-measures ANOVA and the Student-Newman-Keuls *post hoc* test for multiple comparisons. The difference was considered significant if the p value was <0.05.
